# Characterization and Mechanism Prediction of Active Components in Fuganlin Oral Liquid for Respiratory Tract Infections Using UPLC–Q‐TOF–MS and Network Pharmacology

**DOI:** 10.1155/jamc/3075224

**Published:** 2026-02-11

**Authors:** Mengyue Zhang, Feng Han, Mingxuan Yang, Zhishan Ye, Ying Cui, Yuefei Wang, Jing Yang, Xin Chai

**Affiliations:** ^1^ State Key Laboratory of Chinese Medicine Modernization, Institute of Traditional Chinese Medicine, Tianjin University of Traditional Chinese Medicine, Tianjin, 301617, China, tjutcm.edu.cn; ^2^ Research Center of Chinese Materia Medica, Guangzhou Yipinhong Pharmaceutical Co., Ltd., Guangzhou, 510530, Guangdong, China; ^3^ Department of Science and Technology Development, Haihe Laboratory of Modern Chinese Medicine, Tianjin, 301617, China

## Abstract

Fuganlin oral liquid (FOL) has been clinically employed for the treatment of pediatric qi deficiency colds, manifesting symptoms such as fever, cough, asthma, and sore throat. However, the chemical composition and bioactive components of FOL have not been clearly elucidated. In this study, a comprehensive qualitative analysis of FOL was conducted utilizing ultra‐high‐performance liquid chromatography coupled with quadrupole time‐of‐flight mass spectrometry in conjunction with network pharmacology. A total of 124 chemical components were tentatively characterized, comprising flavonoids, phenolic acids, saponins, coumarins, and others. Among these, 43 compounds were unequivocally identified by comparison with authentic reference standards. Furthermore, network pharmacology analysis revealed that the ingredients of FOL exhibited anti‐inflammatory properties and demonstrated potential efficacy in relieving cough and asthma associated with respiratory tract infections. Collectively, this study provides the first comprehensive characterization of the chemical composition in FOL and explores the potential pharmacological mechanisms of its bioactive constituents, thereby providing scientific support for quality control standards and clinical applications.

## 1. Introduction

Respiratory tract infections (RTIs) are predominantly caused by viruses such as influenza, coronavirus (including SARS‐CoV‐2), and respiratory syncytial virus (RSV), as well as bacteria and fungi. These infections affect individuals of all ages, particularly children and those with weakened immune systems, potentially leading to severe acute respiratory syndrome. According to epidemiological data, respiratory infections constitute the leading cause of disease burden when measured by disability‐adjusted life years (DALYs). Recent projections from the Global Burden of Diseases (GBD) study indicate a concerning upward trend, with RTIs incidence rates anticipated to increase by 76.41% by 2050. These infections often trigger oxidative stress reactions and immune system dysfunctions, resulting in further lung tissue damage and hindrance in the recovery of lung functions. Clinical manifestations range from mild symptoms, such as sore throat and nasal congestion, to severe complications, including pneumonia, multiorgan failure, and even fatality [1, 2]. Due to the high infectivity, variability, and incidence rates of RTIs, treatment options based on antiviral medications are limited. Commonly utilized antiviral drugs in Western medicine, such as valacyclovir, remdesivir, and acyclovir, are prone to the development of drug resistance and are associated with various side effects and limited efficacy.

Fuganlin oral liquid (FOL) is a clinically effective compound preparation that comprehensively regulates human immune functions, offering distinct advantages in the prevention and treatment of respiratory viral diseases. Originating from a folk experience prescription, FOL comprises Herba Bidentis Pilosae (Guizhencao, HBP), Flos Chrysanthemi Indici (Yejuhua, FCI), Radix Panacis Quinquefolii (Xiyangshen, RPQ), Radix Astragali (Huangqi, RA), Radix Isatidis (Banlangen, RI), Edodes Lentinus (Xianggu, EL), Bulbus Fritillariae Thunbergii (Zhebeimu, BFT), Herba Ephedrae (Mahuang, HE), Radix Peucedani (Qianhu, RP), and Radix et Rhizoma Glycyrrhizae (Gancao, RRG). According to traditional Chinese medicine (TCM) theory, FOL possesses the ability to clear heat and detoxify, relieve cough and asthma, and enhance qi, thereby alleviating symptoms such as fever, cough, asthma, pharyngeal swelling, and pain associated with qi‐deficiency and wind‐heat colds in children. In FOL, FCI and RI help clear heat and detoxify, while HBP synergistically enhances these effects, reducing fever and sore throat symptoms associated with colds. RPQ and RA act as qi‐benefiting agents for the lungs and spleen, making them suitable for patients with deficiencies, presenting symptoms such as sweating, yellowish complexion, and poor appetite. BFT, HE, and RP serve as adjuvants, aiding in lung relief and alleviating cough, phlegm, and asthma. RRG harmonizes all the herbs present in FOL. Pharmacological studies have suggested that FOL exhibits antipyretic, antitussive, and bacteriostatic effects, particularly against bacteria like *Staphylococcus aureus* and *Staphylococcus albus* in RTIs [3]. However, comprehensive studies on the complete compound profiles of FOL and its therapeutic mechanism for RTIs are scarce.

Recent advancements in high‐resolution mass spectrometers offer enhanced evaluation of TCM constituents due to diversified acquisition methods, faster scanning capabilities, and higher sensitivities [4]. Liquid chromatography–mass spectrometry technology, recognized for its rapid separation ability and ultra‐high sensitivity, is pivotal in analyzing the intricate components of TCM, facilitating the modernization and efficiency of TCM analysis. Network pharmacology, an emerging field in TCM research, aids in elucidating active ingredients, predicting potential targets, and examining the correlation with disease syndromes through the construction of biological network models. This approach facilitates understanding TCM’s multitarget and multipathway mechanisms, providing a theoretical basis for future research and applications [5, 6].

In this study, a rapid and effective chemical characterization method of FOL was established using ultra‐high performance liquid chromatography coupled with quadrupole‐time of flight mass spectrometry (UPLC–Q‐TOF–MS). Subsequently, the molecular mechanism of FOL in treating RTIs was elucidated using network pharmacology analysis approaches. For the first time, the primary chemical components, their attributions, and potential pharmacological effects of FOL were investigated, offering a reference for further research on the quality control, pharmacodynamic clarification, and clinical applications of FOL.

## 2. Materials and Methods

### 2.1. Reagents and Materials

LC‐grade methanol was purchased from Sigma‐Aldrich Inc. (St. Louis, MO, USA). LC‐grade formic acid was obtained from Shanghai Aladdin Biochemical Technology Co. Ltd. (Shanghai, China). Dimethyl sulfoxide (DMSO) was offered by Tianjin Damao Chemical Reagent Factory (Tianjin, China). Analytical grade methanol was purchased from Tianjin Concord Technology Co., Ltd. (Tianjin, China). Water used in all the experiments was purified using the Millipore Milli‐Q system (Milford, MA, USA). FOL (10121002) and HBP (YC220806) were provided by Guangzhou Yipinhong Pharmaceutical Co., Ltd. (Guangdong, China). FCI (13532205002), RA (10212109002), EL (19052207001), and HE (2220207) were obtained from Shaohuatang Traditional Chinese Medicine Co., Ltd. (Anhui, China). RPQ (20220111001) was procured from Guangdong Medicine Pharmaceutical Co., Ltd. (Guangdong, China). RI (20221121) was acquired from Guangdong Tiansheng Pharmaceutical Co., Ltd. (Guangdong, China). BFT (JZT20221214) was purchased from Jointown Group Anguo Traditional Chinese Medicine Co., Ltd. (Hebei, China). RP (Y2021122801) was bought from Gansu Jiuzhou Tianrun Traditional Chinese Medicine Industry Co., Ltd. (Gansu, China). RRG (20221001) was attained from Anhui Hejitang Chinese Medicine Co., Ltd. (Anhui, China).

Reference compounds were obtained from Shanghai Yuanye Bio‐Technology Co., Ltd. (Shanghai, China), including adenosine, sucrose, isochlorogenic acid B, gallic acid, liquiritin, physcion, protocatechuic acid, isoliquiritigenin, fructose, quinic acid, caffeic acid, diosmetin, naringenin, licochalcone A, eupatilin, chlorogenic acid, quercetin, kaempferol‐3‐*O*‐rutinoside, ethyl‐3,4‐dihydroxybenzoate, myricetin, adenosine 3′,5′‐cyclophosphate, imperatorin, azelaic acid, isopimpinellin, astilbin, chrysin, myricetin, curcumin, pinoresinol, coumarin, peimine, peiminine, ononin, emodin, scopolin, and naringin. Besides, isoastragaloside I and formononetin were acquired from Tianjin Yifang Technology Co., Ltd. (Tianjin, China). Tanshinone IIA was bought from Chengdu Ruifensi Biotechnology Co., Ltd. (Chengdu, China). Baicalin was offered from the China Institute for the Control of Pharmaceutical and Biological Products (Beijing, China). Isopsoralen was attained from Shanghai Standard Technology Co., Ltd. (Shanghai, China). Apioside liquiritin was gained from Chengdu Pufeide Biotechnology Co., Ltd. (Chengdu, China). Hypoxanthine was provided by Sigma‐Aldrich Inc. (St. Louis, MO, USA). The purity of these reference compounds was all determined to be above 98% by UPLC analysis.

### 2.2. Preparation of Standard and Sample Solutions

The reference standards were accurately weighed and dissolved in a methanol‐aqueous solution to obtain a mixed reference solution with the following concentrations: 56.00 μg·mL^−1^ isochlorogenic acid B, 45.20 μg·mL^−1^ gallic acid, 48.40 μg·mL^−1^ baicalin, 59.20 μg·mL^−1^ sucrose, 40.00 μg·mL^−1^ isopsoralen, 40.80 μg·mL^−1^ adenosine, 59.60 μg·mL^−1^ formononetin, 50.40 μg·mL^−1^ liquiritin, 44.80 μg·mL^−1^ physcion, 38.40 μg·mL^−1^ fructose, 44.00 μg·mL^−1^ tanshinone IIA, 46.00 μg·mL^−1^ hypoxanthine, 66.00 μg·mL^−1^ quinic acid, 52.00 μg·mL^−1^ caffeic acid, 39.60 μg·mL^−1^ liquiritin apioside, 50.80 μg·mL^−1^ protocatechuic acid, 40.40 μg·mL^−1^ isoliquiritigenin, 41.20 μg·mL^−1^ diosmetin, 38.40 μg·mL^−1^ isoastragaloside I, 44.40 μg·mL^−1^ naringin, 50.80 μg·mL^−1^ kaempferol‐3‐*O*‐rutinoside, 47.60 μg·mL^−1^ naringenin, 39.60 μg·mL^−1^ licochalcone A, 59.60 μg·mL^−1^ isorientin, 46.00 μg·mL^−1^ chlorogenic acid, 40.00 μg·mL^−1^ quercetin, 39.60 μg·mL^−1^ ethyl‐3,4‐dihydroxybenzoate, 34.40 μg·mL^−1^ myricetin, 38.80 μg·mL^−1^ adenosine 3′,5′‐cyclophosphate, 46.00 μg·mL^−1^ imperatorin, 38.80 μg·mL^−1^ azelaic acid, 35.60 μg·mL^−1^ isopimpinellin, 39.20 μg·mL^−1^ astilbin, 38.80 μg·mL^−1^ chrysin, 48.80 μg·mL^−1^ myricetin, 32.00 μg·mL^−1^ curcumin, 42.80 μg·mL^−1^ pinoresinol, 32.00 μg·mL^−1^ coumarin, 49.60 μg·mL^−1^ peimine, 53.20 μg·mL^−1^ peiminine, 34.40 μg·mL^−1^ formononetin, 39.60 μg·mL^−1^ emodin, and 44.80 μg·mL^−1^ scopolin. All solutions were stored at 4°C when not in use.

To prepare the FOL sample solution, 1 mL of FOL was measured precisely and transferred to a 5‐mL volumetric flask. The volume was fixed to scale with methanol, and the mixture was shaken and precipitated in an ice‐water bath for 30 min. The mixture was then vortexed and centrifuged at 12,000 rpm for 15 min to obtain the supernatant as the FOL sample solution. HBP (10 g), RA (10 g), RI (10 g), EL (10 g), RRG (10 g), RP (10 g), and FCI (10 g) were respectively extracted with 70 mL of water by refluxing for two 2 h cycles. RPQ (10 g) was accurately weighed and extracted with 70 mL of 70% ethanol solution by heating and refluxing for two 2 h cycles. HE (10 g) and BFT (10 g) were weighed, added to 70 mL of 80% ethanol solution, and refluxed twice for 1 h each time. Then, 1 mL of the decoction of each single herb was measured and processed using the same procedures as the FOL solution to obtain the test solutions.

### 2.3. UPLC–Q‐TOF–MS Analysis

Chromatographic separation was performed on an ACQUITY UPLC I‐Class, equipped with an Agilent Zorbax SB‐C18 column (4.6 mm × 100 mm, 1.8 micron; Agilent Technologies, USA) at a temperature of 30°C. The mobile phase system consisted of 0.1% formic acid aqueous solution (v/v, A) and methanol (B), with the following optimized gradient program: 5%–80% B in 0–26 min and 80%–95% B in 26–36 min. The flow rate was maintained at 0.3 mL/min, and the injection volume was set at 3 μL.

High‐resolution MS data were acquired by a Vion^TM^ IM‐QTOF mass spectrometer coupled with a Z‐spray^TM^ electrospray ionization (ESI) source (Waters, Milford, MA, USA). The HDMS^E^ acquisition method was employed to collect data in both positive and negative modes. The ESI source parameters were set as follows: capillary voltage, 1.5 kV; cone voltage, 40 V; source temperature, 120°C; desolvation temperature, 500°C; cone gas flow rate (N_2_), 50 L/h; desolvation gas flow rate (N_2_), 800 L/h. The mass analyzer scanned over a mass range of 100–1500 Da in full scan mode, with a scan time of 0.3 s under the low collision energy of 6 eV. The MS/MS experiments were performed under the optimized ramp collision energy (RCE) ranging from 20 to 60 eV across the same mass range.

### 2.4. UNIFI Data Processing Method

The compounds derived from the 10 herbs in FOL were systematically compiled by conducting extensive searches across various databases, including China National Knowledge Infrastructure (CNKI), PubMed, PubChem, ChemSpider, and other relevant sources. A UNIFI‐compatible holographic database was established by thoroughly reviewing research on the chemical components of each herb in FOL, summarizing details such as compound names, molecular formulas, molecular weights, and structure formulas. UNIFI was then utilized to access the self‐built and shared database. Compound identification was achieved through database retrieval, comparison with reference substances, analysis of secondary mass spectrometry fragmentation patterns, and comparison with reference data. The chemical components in FOL were classified according to the mass spectrometry data obtained from each individual herb.

### 2.5. Network Pharmacology Analysis

The SMILES structures of the compounds identified through mass spectrometry analysis were downloaded from PubChem (https://pubchem.ncbi.nlm.nih.gov/) and imported into the SwissTargetPrediction platform (https://www.swisstargetprediction.ch/), with the species parameter set as “*Homo sapiens*” for target prediction. A candidate target library for the chemical components in FOL was constructed by aggregating targets from the Traditional Chinese Medicine Systems Pharmacology Database and Analysis Platform (TCMSP, https://www.tcmsp-e.com/) and the Integrative Pharmacology‐based Research Platform of Traditional Chinese Medicine (TCMIP, https://www.tcmip.cn/TCMIP/index.php/) database while removing any duplicates. Genes associated with RTIs and key bioactivities, including anti‐inflammatory effects and relieving cough and asthma, were retrieved from the GeneCards database (https://www.genecards.org/), with the limit of “Relevance score” > 10. The common targets were then imported into the STRING database (https://string-db.org/) to construct a protein–protein interaction (PPI) network. A “Chinese herbal medicines (CHMs)–preparation–compounds–targets–bioactivities–RTIs” network was established to illustrate the relationships among these elements using Cytoscape 3.7.2. Gene Ontology (GO) and Kyoto Encyclopedia of Genes and Genomes (KEGG) enrichment analyses were performed using the DAVID database (https://david.ncifcrf.gov/).

## 3. Results and Discussion

### 3.1. Characterization of Chemical Constituents in FOL by UPLC–Q‐TOF–MS Combined With UNIFI Software

To ensure the comprehensive and accurate compound identification, a self‐built database was developed using UNIFI software. A total of 575 compounds were retrieved from the 10 herbs of FOL, including their names, molecular formulas, molecular weights, chemical structures, and so on. A UPLC–Q‐TOF–MS method was established to characterize the chemical constituents in FOL, with the corresponding base peak intensity (BPI) chromatograms in both positive and negative ion modes displayed in Figures 1(a) and 1(b). Through analysis of retention times, accurate mass measurements, and fragmentation patterns, 124 chemical constituents were preliminarily identified, as summarized in Table 1, including 50 flavonoids, 16 organic acids, 13 saponins, 16 coumarins, and 29 other components. Notably, 43 of these compounds were confirmed by comparison with reference standards, as shown in Figures 1(c) and 1(d).

FIGURE 1Base peak intensity chromatograms of FOL in positive (a) and negative (b) ion modes, and mixed standards solution in positive (c) and negative (d) ion modes, by UPLC–Q‐TOF–MS.

**Figure:**
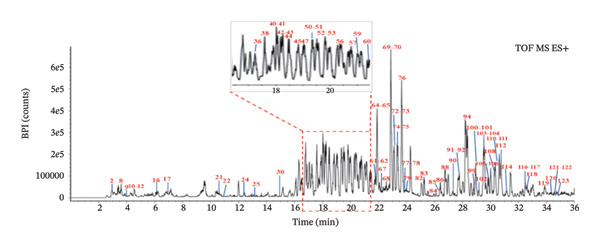
(a)

**Figure:**
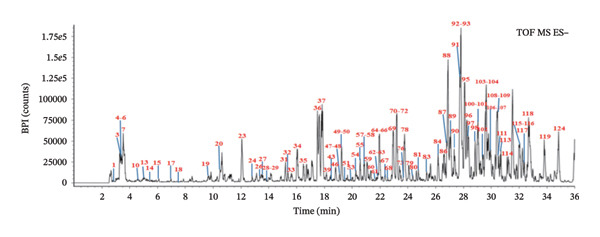
(b)

**Figure:**
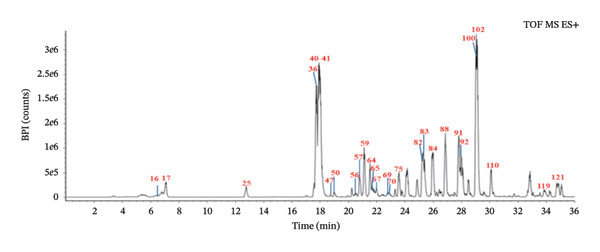
(c)

**Figure:**
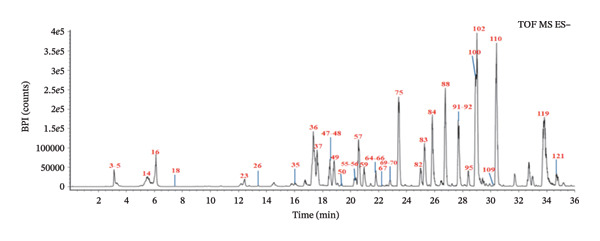
(d)

**TABLE 1:** Characterization of chemical constituents in FOL by UPLC–Q‐TOF–MS.

No.	*t*_*R*_ (min)	Formula	Fragment ions in positive mode (*m/z*)	Fragment ions in negative mode (*m/z*)	Identification	Source
1	2.88	C_6_H_14_N_4_O_2_	—	173.1032, 130.0971	Arginine [7]	HBP, RRG, RI, RA, RP, EL, RPQ
2	2.97	C_10_H_14_N_2_O_6_	259.0911, 241.0813	—	3‐Methyluridine	HBP, RI, RA, RP, EL, RPQ, FCI
3^∗^	3.33	C_12_H_22_O_11_	—	341.1078, 179.0531, 161.0433	Sucrose [8]	HBP, HE, RRG, RI, RA, RP, EL, RPQ, FCI, BFT
4^∗^	3.34	C_6_H_12_O_6_	—	179.0548, 143.0341	Fructose	HBP, HE, RRG, RI, RA, RP, EL, RPQ, FCI, BFT
5^∗^	3.34	C_7_H_12_O_6_	—	191.0548, 129.0181, 111.0079	Quinic acid [9]	HBP, RPQ, FCI
6	3.37	C_6_H_12_N_2_O_4_	—	175.0711, 147.0259	Serylalanine [10]	RA, RP, EL, RPQ, BFT
7	3.47	C_5_H_10_O_5_	—	149.0444, 95.9338	Arabinose	HBP, RRG, RI, EL, RPQ
8	3.57	C_12_H_16_N_2_O_4_	253.1191, 217.1267	—	Tyrosylalanine	RRG, RI, RP, RPQ
9	4.08	C_15_H_16_O_3_	245.1172, 283.0723	—	Osthole [11]	RRG
10	4.55	C_9_H_11_N_5_O_4_	254.0869, 218.1369	252.0732, 134.0462	Eritadenine [12]	EL
11	4.58	C_5_H_7_NOS	130.0334, 70.0658	—	(*R*,*S*)‐goitrin [13]	RI
12	4.59	C_16_H_16_O_3_	257.1182, 254.0868, 238.1171	—	Ichthyothereol acetate	HBP
13	5.04	C_5_H_7_NO_3_	—	128.0342, 85.0283	Pyroglutamic acid [14]	HBP, RRG, EL, RPQ, FCI, BFT
14^∗^	5.44	C_10_H_13_N_5_O_4_	—	135.0305	Hypoxanthine	HBP, RRG, RA, RP, EL
15	6.06	C_9_H_11_NO_3_	—	180.0650, 163.0370	Tyrosine [7]	RA, RP, EL, FCI
16^∗^	6.23	C_10_H_13_N_5_O_4_	268.1026, 136.0600, 119.0328	—	Adenosine [15]	HBP, HE, RRG, RI, RA, RP, EL, RPQ, FCI, BFT
17^∗^	6.93	C_10_H_12_N_5_O_6_P	330.0583, 312.1433, 136.0638	328.0430, 134.0468	Adenosine 3′,5′‐cyclophosphate [7]	RRG, RI, RA, RP, EL, FCI
18^∗^	7.52	C_7_H_6_O_5_	—	169.0130, 125.0249	Gallic acid [7]	HBP, RRG
19	9.66	C_17_H_20_O_10_	—	383.0974, 339.1509, 311.1211	Eleutheroside B1	RP
20	10.44	C_21_H_26_O_13_	—	485.1340, 427.1775	Hymexelsin [16]	RP
21	10.72	C_10_H_15_NO	166.1240, 148.1132	—	Ephedrine hydrochloride [14]	HE
22	10.96	C_10_H_15_NO	166.1228, 148.1136	—	Pseudoephedrine hydrochloride [14]	HE
23^∗^	12.04	C_7_H_6_O_4_	—	153.0192, 109.0289, 91.0184	Protocatechuic acid [7]	HBP, HE, RI, EL
24	12.66	C_10_H_8_O_4_	193.0493	191.0343	7‐Methoxy‐6‐hydroxycoumarin or its isomer	HBP
25^∗^	13.03	C_16_H_18_O_9_	355.1004, 193.0534, 178.0301	—	Scopolin [16]	HBP, RP
26^∗^	13.31	C_16_H_18_O_9_	—	353.0871, 191.0549, 161.0238	Chlorogenic acid [7]	HBP, RPQ, FCI
27	13.62	C_19_H_20_O_6_	—	343.1175, 295.0583, 205.0706	*D*‐Laserpitin	RP
28	13.85	C_30_H_36_O_9_	—	539.2323, 507.1916, 445.2044	Sesquispanol B	HE
29	13.89	C_15_H_14_O_7_	—	305.0683, 287.1535	Epigallocatechin [17]	HBP, RI
30	14.83	C_10_H_9_NO_2_	176.0697, 162.0537	—	3‐Hydroxyacetyl indole [18]	HBP
31	15.05	C_15_H_12_O_7_	—	303.0500, 225.1120	7,8,3′,4′‐Tetrahydroxyflavonol or its isomer [19]	HBP
32	15.33	C_15_H_20_O_9_	—	343.1019, 315.0900, 197.0446	Syringic acid‐4‐*O-α*‐L‐rhamnoside [20]	HBP
33	15.60	C_28_H_32_O_17_	—	639.1545, 621.2387	Astragaloside	RA
34	15.86	C_27_H_30_O_17_	—	625.1424, 551.2130, 389.1629	Quercetin‐3‐*O-β*‐D‐galactose‐7‐*O-β*‐*D*‐Glucose [21]	HBP
35^∗^	16.30	C_9_H_8_O_4_	—	179.0340, 161.0229, 135.0442	Caffeic acid [7]	HBP, HE, RA, RP
36^∗^	17.46	C_26_H_30_O_13_	551.1773, 533.2205, 521.2662	549.1609, 417.1187, 255.0649	Liquiritin apioside [22]	RRG
37^∗^	17.78	C_21_H_22_O_9_	—	417.1185, 255.0649	Liquiritin [22]	RRG
38	17.80	C_11_H_10_O_5_	223.0597, 207.0752	—	Isofraxidin [23]	HE, RP
39	17.98	C_20_H_24_O_10_	—	423.1292, 405.1550, 375.1641	Apterin [16]	RP
40^∗^	18.06	C_27_H_45_NO_3_	432.3466, 414.3357, 396.3042	—	Peimine [15]	BFT
41^∗^	18.09	C_27_H_45_NO_3_	430.3431, 412.3203, 394.2888	—	Peiminine [15]	BFT
42	18.36	C_30_H_34_O_13_	603.2063, 547.1463	—	Sesquiterpene	HE, FCI
43	18.44	C_27_H_30_O_16_	611.1636, 465.1274, 303.0548	609.1461, 301.0338	Quercetin‐3‐*O*‐robinobioside [7]	HBP, HE, RP
44	18.51	C_16_H_22_O_8_	343.1388, 365.1215	—	Praeruptorin I [24]	RP
45	18.72	C_21_H_20_O_11_	449.1073, 303.0559	—	Quercetin‐7‐*O*‐rhamnoside [25]	HBP, HE, FCI
46	18.75	C_21_H_20_O_11_	—	447.0929, 431.1957, 415.1036, 253.0488	5,8,4′‐Trihydroxyflavone‐7‐*O-β*‐D‐glucoside or its isomer [19]	HBP
47^∗^	18.92	C_25_H_24_O_12_	517.1344, 355.1041	515.1189, 353.0876	Isochlorogenic acid B [8]	HBP
48^∗^	18.98	C_21_H_22_O_11_	—	449.1080, 287.0548	Astilbin	HBP, RA, FCI
49^∗^	19.14	C_27_H_32_O_14_	—	579.1712, 433.1414, 271.0591	Naringin [7]	RI, FCI
50^∗^	19.21	C_21_H_20_O_12_	465.1021, 303.0488	463.0880, 301.0336	Myricetrin	HBP, HE, RA, FCI
51	19.38	C_16_H_12_O_7_	317.0646, 302.0704	315.0491, 279.0243	5,7,3′,4′‐Tetrahydroxy‐3‐methoxyflavone or its isomer [26]	HBP
52	19.80	C_14_H_14_O_4_	247.0953, 229.0840	—	Marmesine [16]	RP
53	19.82	C_23_H_24_O_13_	509.1290, 347.0754	507.1141, 345.0600	Axillaroside	HBP
54	20.26	C_15_H_14_O_5_	—	273.0746, 263.0909, 221.1161	Afzelechin	HE
55^∗^	20.46	C_9_H_10_O_4_	—	181.0495, 137.0595	Ethyl‐3,4‐dihydroxybenzoate	HE, RI
56^∗^	20.47	C_9_H_6_O_2_	147.0436, 119.0521, 103.0348	—	Coumarin [7]	HBP
57^∗^	20.78	C_27_H_30_O_15_	595.1664, 449.1104	593.1516, 285.0404, 163.0032, 151.0037	Kaempferol‐3‐*O*‐rutinoside [7]	HBP, HE
58	20.88	C_20_H_20_O_7_	—	371.1131, 353.1019, 325.1579	Sinensetin [27]	RI
59^∗^	21.03	C_22_H_22_O_9_	431.1337, 269.0797, 241.0515	429.1170, 475.1263	Ononin [28]	RA
60	21.39	C_17_H_14_O_7_	331.0800, 316.0565, 301.0331	329.0658, 314.0416, 299.0182	3,3‐Dimethoxyquercetin [29]	HBP, FCI
61	21.65	C_23_H_24_O_12_	493.1339, 457.2337, 331.0802	491.1189, 455.1957	3,5‐Dihydroxy‐3′,5′‐dimethoxyflavone‐7‐*O-β*‐D‐glucopyranoside or its isomer [19]	HBP
62	21.73	C_24_H_26_O_13_	523.1445, 361.0910	521.1305, 506.1305, 462.2358, 300.0259	5,3′‐Dihydroxy‐3,6,4′‐trimethoxy‐7‐*O-β*‐D‐glucopyranoside flavone or its isomer [30]	HBP
63	21.76	C_28_H_36_O_15_	—	611.1987, 563.1408	Neohesperidin dihydrochalcone or its isomer [21]	HBP
64^∗^	21.86	C_21_H_18_O_11_	447.0920, 271.0587	445.0772, 427.1573, 269.0457	Baicalin [31]	HBP, RA, FCI
65^∗^	21.90	C_15_H_10_O_7_	303.0489, 285.0392	301.0341, 273.0384, 151.0008	Quercetin [7]	HBP, RRG, RA
66^∗^	21.93	C_9_H_16_O_4_	—	187.0963, 169.0862, 125.0994	Azelaic acid	HBP, HE, RRG, RI, RA, RP, EL, RPQ, FCI, BFT
67^∗^	22.20	C_15_H_10_O_8_	319.0432, 301.2224, 273.1129	317.0287, 243.1232	Myricetin [17]	RA, RP, FCI
68	22.51	C_28_H_32_O_16_	625.1769, 581.1875, 463.1233	623.1639, 461.1095, 299.0555	Complanatuside [32]	RA
69^∗^	22.70	C_20_H_22_O_6_	359.1491	357.1332, 313.0720, 269.0441	Pinoresinol	HBP
70^∗^	22.84	C_15_H_12_O_5_	273.0747, 255.0280, 227.0343	271.0599	Naringenin [9]	HBP, RRG, RI, RA, RP
71	23.04	C_18_H_34_O_10_	—	409.2072, 345.2261, 277.1638	Heptanyl‐2‐*O*‐*β*‐xylofuranosyl‐(1 ⟶ 6)‐*β*‐gluco pyranoside [33]	HBP
72	23.06	C_16_H_12_O_5_	285.0663	283.0634, 268.0456	Calycosin [34]	RA
73	23.28	C_28_H_32_O_14_	593.1843, 285.0733	637.1857, 591.1721	Buddleoside	FCI
74	23.49	C_12_H_8_O_4_	217.0481, 202.0234	—	6‐Methoxyangeletin [16]	RP
75^∗^	23.51	C_11_H_6_O_3_	187.0384, 131.0516	—	Isopsoralen [16]	RP
76	23.53	C_17_H_14_O_6_	315.0852, 300.0628	313.0711, 299.1821	Odoratin	RA
77	23.71	C_30_H_48_O_2_	441.3729, 423.3620	—	Betulone	RRG
78	23.74	C_48_H_82_O_18_	587.4154, 423.3604, 405.3490	992.5599	Ginsenoside Re [35]	RPQ
79	24.01	C_42_H_72_O_14_	423.3632, 405.3531	845.4907	Ginsenoside Rg1 [35]	RPQ
80	24.23	C_18_H_26_O_4_	—	305.1741, 293.1768, 267.1511	Phthalic acid butyl isohexyl ester	HBP
81	24.75	C_20_H_32_N_2_O_3_	—	347.2368, 311.1690, 283.0588	*L*‐(−)Ephedrine hemihydrate	HE
82^∗^	25.19	C_13_H_10_O_5_	247.0586, 269.0417	—	Isopimpinellin [16]	HBP, RP
83^∗^	25.35	C_15_H_12_O_4_	257.0794, 239.0690	255.0649, 135.0075	Isoliquiritigenin [36]	HBP, RRG
84^∗^	25.83	C_16_H_12_O_6_	301.0700, 286.0466, 258.0839	299.0549, 284.0312	Diosmetin [9]	HBP, RRG
85	26.31	C_24_H_28_O_7_	429.1909, 411.1795, 349.6519	—	Peucedanum coumarin H [37]	RP
86	26.44	C_48_H_72_O_21_	985.4700, 809.4368, 615.3908, 453.3313	983.4495, 821.4869, 645.3643	Licoricesaponin A3 [34]	RRG
87	26.66	C_20_H_30_N_6_O_12_S_2_	—	609.1238, 574.2778, 560.2779, 544.2809	Oxidized glutathione	FCI
88^∗^	26.77	C_16_H_12_O_4_	269.0796, 253.0506, 237.0552, 225.0545, 213.0926	267.0653, 252.0394, 223.0371	Formononetin [7]	RRG, RA
89	26.93	C_25_H_28_O_4_	—	391.1878, 374.0559, 346.1467	Glabrol	HE, RRG
90	27.33	C_42_H_72_O_14_	801.5025	799.4859, 653.4283, 635.4154	Pseudoginsenoside F11 [35]	RPQ
91^∗^	27.61	C_21_H_20_O_6_	369.1313, 351.2135	367.1177, 352.0988	Curcumin	RRG, FCI
92^∗^	27.68	C_18_H_16_O_7_	345.0964, 331.1876, 293.2064, 276.2025	343.0809, 325.2297, 291.1958	Eupatilin [38]	HBP, RA
93	27.77	C_42_H_64_O_16_	—	823.4121, 805.3836, 761.4147	Uralsaponin C	RRG
94	27.89	C_30_H_24_O_10_	545.1433, 471.3413	—	Mahuannin A	HE
95^∗^	28.17	C_16_H_14_O_4_	—	269.0819, 200.1346	Imperatorin [27]	HBP, RRG, RA, RP
96	28.31	C_47_H_80_O_19_	—	947.5236, 785.4697	Vietnamese ginsenoside R6 [35]	RPQ
97	28.41	C_21_H_22_O_5_	—	353.1388, 322.0016, 293.1781	Gancaonin I [39]	RRG
98	28.75	C_20_H_18_O_6_	—	353.1023, 325.1973, 297.1497	Glycyrrhflavone	RRG
99	28.80	C_26_H_43_NO_6_	466.3152, 439.3562, 267.0663	—	Glycocholic acid	BFT
100^∗^	28.85	C_15_H_10_O_4_	255.0639, 241.0464, 213.0542	253.0493, 209.0619, 181.1667	Chrysin [31]	RRG, FCI
101	28.96	C_42_H_62_O_17_	839.4093, 469.3315	837.3930, 819.3813, 775.3913	Licoricesaponin G2 [34]	RRG
102^∗^	29.19	C_16_H_12_O_5_	285.0748, 270.0511	283.0598, 268.0360, 240.0409	Physcion [40]	RRG, RA, FCI
103	29.47	C_42_H_65_NO_16_	823.4055, 453.3342	821.4014	Ammonium glycyrrhizinate	RRG
104	29.64	C_54_H_92_O_23_	1109.6065, 767.4885	1153.6117	Ginsenoside Rb1 [35]	RPQ
105	29.69	C_30_H_50_O_2_	443.3878, 425.3773	—	Uvaol	BFT
106	29.70	C_54_H_92_O_23_	1109.6148, 1047.8293	1107.5977, 961.5375, 799.4889	Yesanchinoside E [41]	RPQ
107	29.85	C_53_H_90_O_22_	—	1077.5869, 799.3748	Ginsenoside Rb3 [35]	RPQ
108	30.29	C_22_H_22_O_6_	383.1486, 365.2308, 351.2484	381.1332, 325.1837	Glycyrin	RRG
109^∗^	30.30	C_19_H_18_O_3_	—	293.1203, 249.1875, 221.1538	Tanshinone IIA [42]	RRG, RP
110^∗^	30.43	C_21_H_22_O_4_	339.1617, 322.1288, 308.1723, 283.1219	—	Licorice chalcone A [43]	HBP, RRG, FCI
111	30.44	C_48_H_76_O_19_	957.5108, 811.4505	955.4918, 793.4439, 731.4329, 613.3707, 569.3810	Ginsenoside Ro [44]	RPQ
112	30.64	C_14_H_10_O_3_	227.0688, 199.0770	—	Arnocoumarin [16]	RP
113	30.75	C_55_H_92_O_23_	—	1119.5946, 1077.5862, 942.5017, 808.4215	Ginsenoside Rs1 [35]	RPQ
114	31.19	C_48_H_78_O_18_	943.5311, 925.5214, 797.4712, 635.4168	941.5126, 795.4557	Soyasaponin I [28]	RA
115	32.13	C_43_H_70_O_15_	—	825.4615, 765.4498	Astragaloside II [28]	RA
116	32.46	C_41_H_68_O_14_	807.4429, 587.3907	829.4645	Astragaloside IV [45]	RA
117	32.46	C_15_H_22_O_2_	235.1679	233.1533	10‐Oxo‐isodauc‐3‐en‐15‐al [46]	HBP
118	32.65	C_41_H_68_O_14_	785.4702, 767.4594, 749.4476, 587.3945	783.4553, 621.3978	Astragaloside III [28]	RA
119^∗^	33.94	C_15_H_10_O_5_	271.0592	269.0446, 241.0495, 213.0542	Emodin [7]	RRG, FCI
120	34.47	C_41_H_77_NO_9_	728.5711, 711.1719, 675.3778	—	Cerebroside B	HBP
121^∗^	34.66	C_45_H_72_O_16_	869.4938, 851.4768, 689.4259, 671.4128	—	Isoastragaloside I [7]	RA
122	34.75	C_43_H_70_O_15_	827.4817, 809.4702, 791.4605	—	Isoastragaloside II [7]	RA
123	34.85	C_20_H_30_O_3_	319.2251, 308.2184	—	Ineketone	RI
124	34.95	C_48_H_82_O_17_	—	929.5454, 783.4873	Vinaginsenoside R3 [44]	RPQ

^∗^Compared with the reference standard.

Abbreviations: BFT, Bulbus Fritillariae Thunbergii; EL, Edodes Lentinus; FCI, Flos Chrysanthemi Indici; HBP, Herba Bidentis Pilosae; HE, Herba Ephedrae; RA, Radix Astragali; RI, Radix Isatidis; RP, Radix Peucedani; RPQ, Radix Panacis Quinquefolii; RRG, Radix et Rhizoma Glycyrrhizae.

Flavonoids, primarily derived from HBP and RA, are known for their significant pharmacological functions, including anti‐inflammatory effects, blood pressure regulation, hypolipidemic properties, liver protection, and cardiovascular benefits [47–50]. For instance, compound 57, with a relative molecular mass of 594.1595, exhibited a quasi‐molecular ion of [M − H]^−^ at *m/z* 593.1516 in negative ion mode, along with a fragment ion at *m/z* 285.0404 [M − H − C_12_H_20_O_9_]^−^, resulting from the loss of a rutinose residue under high‐energy collision. The flavonoid aglycone underwent a retro Diels–Alder reaction, producing fragment ions at *m/z* 163.0032 [M − H − C_12_H_20_O_9_ − C_7_H_6_O_2_]^−^ and *m/z* 151.0037 [M − H − C_12_H_20_O_9_ − C_8_H_6_O_2_]^−^. Therefore, compound 57 was conclusively identified as kaempferol‐3‐*O*‐rutinoside based on the second mass spectrum and comparison with reference substance.

Organic acids are recognized for their beneficial properties, including antioxidant, anti‐inflammatory, antibacterial, antithrombotic, and neuroprotective effects [51–53]. In negative ion mode, organic acids typically exhibit a high‐abundance quasi‐molecular ion [M − H]^−^ peak, and their fragmentation pathways often produce small molecules such as CO_2_, CO, and H_2_O, which indicates the presence of carboxyl, carbonyl, or hydroxyl functional groups. For example, in negative ion mode, protocatechuic acid (Compound 23) displayed a quasi‐molecular ion [M − H]^−^ peak at *m/z* 153.0192, with characteristic fragment ions of [M − H − CO_2_]^−^ at *m/z* 109.0289 and [M − H − H_2_O − CO_2_]^−^ at *m/z* 91.0184, corresponding to the loss of CO_2_ and H_2_O, respectively.

Coumarins, associated with various pharmacological activities including antiviral, anticancer, antiosteoporosis, and anticoagulant effects [54–56], are primarily derived from HBP and RP in FOL. The parent structure of coumarin generally features multiple oxygen atoms and hydroxyl groups attached to an aromatic ring, which can undergo ring‐opening reactions under alkaline conditions. In mass spectrometry, coumarins predominantly lose neutral small molecule fragments, such as carbonyl, hydroxyl or H_2_O, methyl or methoxy, and alkyl chains. For example, in positive ion mode, coumarin (Compound 56) was characterized by the presence of the quasi‐molecular ion at *m/z* 147.0436 [M + H]^+^, with fragment ions at *m/z* 119.0521 [M + H − CO]^+^ and *m/z* 103.0348 [M + H − CO_2_]^+^.

Saponins, primarily sourced from RPQ and RRG, are largely composed of triterpenoid saponins in RRG, whose parent nucleus structure is linked to two glucuronic acid residues [57]. During mass spectrometry analysis, the cleavage of glycosidic bonds typically generates characteristic fragments, such as [M + H − GlcA]^+^ and [M + H − 2GlcA]^+^. For instance, compound 86 exhibited a molecular weight of 984.4568, with a [M + H]^+^ ion observed at *m/z* 985.4700 and a [M − H]^−^ ion at *m/z* 983.4495. In the positive ion mode, the secondary mass spectrum revealed prominent peaks at *m/z* 809.4368 [M + H − GlcA]^+^, *m/z* 615.3908 [M + H − 2GlcA − H_2_O]^+^, and *m/z* 453.3313 [M + H − 2GlcA − Glc − H_2_O]^+^, confirming the presence of two glucuronic acid and a glucose moieties in the structure. In the negative ion mode, the quasi‐molecular ion underwent sequential losses, first eliminating a glucose molecule to yield a fragment ion at *m/z* 821.4869 [M − H − Glc]^−^, followed by the removal of a glucuronic acid moiety to produce a fragment ion at *m/z* 645.3643 [M − H − Glc − GlcA]^−^. Based on these fragmentation patterns and spectral data, compound 86 was identified as licoricesaponin A3.

### 3.2. Network Pharmacology

#### 3.2.1. Target Acquisition for FOL and RTIs

**FIGURE 2:**
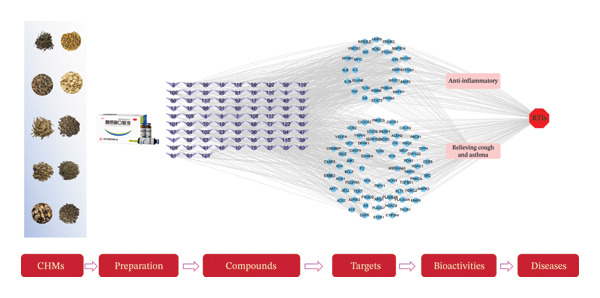
The network of “CHMs–preparation–compounds–targets–bioactivities–RTIs”.

Based on the identified components from FOL, a total of 836 potential targets were retrieved from the SwissTargetsPrediction database. Additionally, 2045 targets associated with RTIs, along with 66 targets linked to anti‐inflammatory activity and 385 targets related to relieving cough and asthma, were obtained from the GeneCards database. Venny 2.1.0 software was used to capture the intersection of FOL‐related and disease‐related targets, resulting in 265 common targets. Further refinement revealed 28 overlapping targets among FOL compounds, RTIs, and anti‐inflammatory activity, as well as 92 shared targets among FOL compounds, RTIs, and cough/asthma relief after merging and removing duplicates. The herbs in FOL, the identified components, the common targets, and their associated biological activities were imported into Cytoscape 3.7.2 to construct the “CHMs–preparation–compounds–targets–bioactivities–RTIs” network, as depicted in Figure 2. The results indicated that FOL compounds exert anti‐RTIs effects by modulating key proteins such as STAT3, TNF, PTGS2, and JUN. Specifically, caffeic acid exhibited anti‐inflammatory effects by targeting STAT3, PTGS1, ELANE, ALOX5, MIF, and TLR4. Quercetin contributed to cough and asthma relief by interacting with PARP1, MMP3, and MMP9. In addition, eupatilin played a significant role in both anti‐inflammatory effect and alleviation of cough and asthma by regulating ALOX5, MPP3, MPP9, MPO, NOS2, and PTGS2. In summary, FOL exerts its therapeutic effects against RTIs through a multicomponent and multitarget approach, highlighting its potential as a comprehensive treatment strategy.

#### 3.2.2. Construction of PPI Networks

**FIGURE 3:**
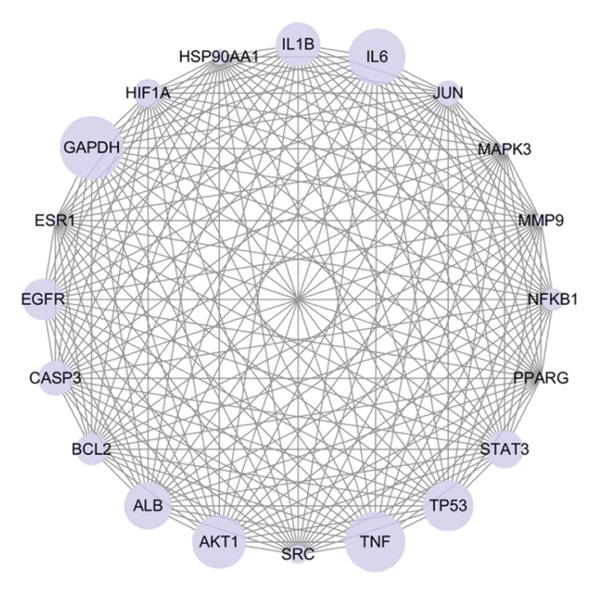
PPI network of top 20 core targets.

The STRING database was utilized to analyze PPIs among the common targets. As illustrated in Figure 3, a PPI network map of FOL’s potential targets against RTIs was constructed, highlighting the top 20 targets ranked by degree value, where GAPDH, TNF, IL6, AKT1, TP53, ALB, IL1*β*, EGFR, STAT3, CASP3, BCL2, HIF1A, JUN, NFκB1, SRC, HSP90AA1, MMP9, MAPK3, ESR1, and PPARG emerged as core targets. Notably, AKT1 phosphorylation plays a pivotal role in regulation processes such as protein synthesis, angiogenesis, cell proliferation, metabolism, and migration. Dominant‐negative AKT1 mutants have been proven to exert a considerable inhibitory effect on viral RNA expression, reduce viral capsid protein expression, and decrease viral release [58]. IL‐6 and TNF are pivotal in immune homeostasis and inflammatory regulation [59]. Additionally, EGFR mediates airway epithelial cell (AEC) repair by upregulating BCL‐2 expression and modulating the Bax/BCL‐2 ratio to inhibit apoptosis. Through PI3K pathway regulation, EGFR influences T‐cell and B‐cell differentiation, potentially alleviating respiratory diseases [60]. These findings suggest the active components in FOL may alleviate RTIs by modulating key proteins, including AKT1, EGFR, and TNF.

#### 3.2.3. GO Function Analysis

To elucidate the molecular mechanisms underlying FOL’s therapeutic effects against RTIs, a GO analysis was performed on the intersected genes using the DAVID database. As demonstrated in Figure 4(a), the enriched items of biological process (BP) include phosphorylation, protein phosphorylation, negative regulation of apoptotic process, positive regulation of MAPK cascade, and positive regulation of protein kinase B signaling. Cellular component (CC) analysis revealed enrichment in receptor complex, plasma membrane, cytoplasm, extracellular region, and cytosol. Molecular function (MF) enrichment results were predominantly associated with enzyme binding, protein kinase activity, and identical protein binding. The GO analysis suggests that the active compounds in FOL may modulate enzyme binding and protein kinase activity by targeting cellular structures, thereby regulating critical signaling pathways involved in inflammation suppression and respiratory symptom relief.

FIGURE 4GO function analysis (a) and KEGG enrichment analysis (b) of the key targets of FOL in the treatment of RTIs.

**Figure:**
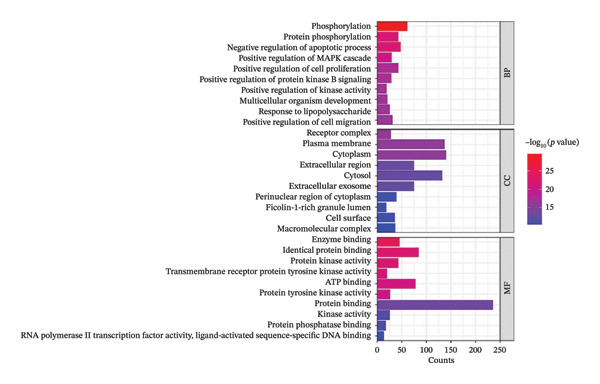
(a)

**Figure:**
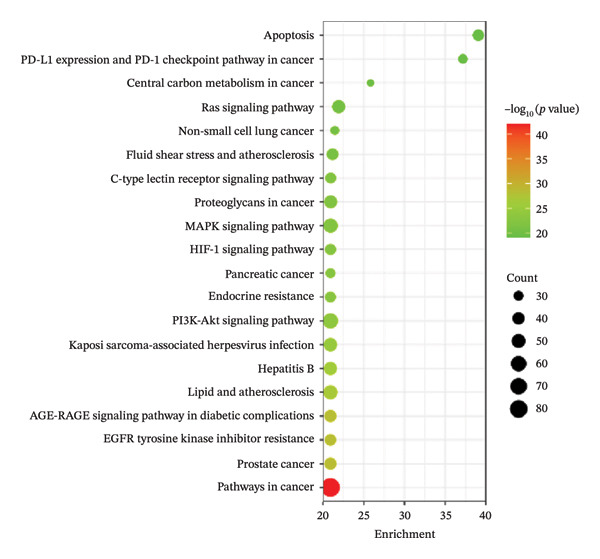
(b)

#### 3.2.4. KEGG Enrichment Analysis

KEGG pathway enrichment was conducted on the core targets, as shown in Figure 4(b). This analysis revealed pathways related to apoptosis; PD‐L1 expression and the PD‐1 checkpoint pathway in cancer; central carbon metabolism in cancer; the Ras signaling pathway; nonsmall cell lung cancer; fluid shear stress and atherosclerosis; the C‐type lectin receptor signaling pathway; and the PI3K‐Akt signaling pathway. The PI3K/Akt signaling pathway plays dual roles in both virus entry and host immune response. Previous studies have established its crucial function in inflammatory regulation through NF‐*κ*B‐mediated control of inflammatory factor expression. The activation of the PI3K/Akt signaling pathway contributes to both inflammatory response and oxidative stress, supporting viral replication via inhibition of premature apoptosis. These findings suggest that PI3K/Akt signaling pathway inhibitors may represent promising therapeutic candidates for viral infections [61].

## 4. Conclusion

In this study, we employed UPLC–Q‐TOF–MS to profile the chemical constituents of FOL, thereby identifying a total of 124 chemical constituents, including 50 flavonoids, 16 organic acids, 13 saponins, 16 coumarins, and 29 other compounds, with 43 authenticated using reference standards. Network pharmacology analysis indicated that the therapeutic effects of FOL against RTIs are mediated through anti‐inflammatory and antitussive activities, primarily via modulation of AGE–RAGE and PI3K/Akt signaling pathways. In summary, this study provides both chemical and mechanistic insights into FOL’s therapeutic potential against RTIs, establishing a foundation for future research.

## Funding

This work was supported by grants from the Science and Technology Program of Tianjin (24ZYJDSS00300) and Special Project for Technological Innovation in New Productive Forces of Modern Chinese Medicines (24ZXZKSY00010).

## Conflicts of Interest

The authors declare no conflicts of interest.

## Supporting Information

Figure S1: The mass spectrum base peak intensity chromatograms of individual herbs from FOL. Table S1: The intersection targets of components in FOL and RTIs. Table S2: The intersection targets of components‐RTIs‐anti‐inflammatory. Table S3: The intersection targets of components‐RTIs‐relieving cough and asthma. Table S4: Top 20 key targets and degree of FOL in the treatment of RTIs.

## Supporting information


**Supporting Information** Additional supporting information can be found online in the Supporting Information section.

## Data Availability

The data used to support the findings of this study are included within the article and the supporting information files.
