# Supplementary material for: Characterization and Mechanism Prediction of Active Components in Fuganlin Oral Liquid for Respiratory Tract Infections Using UPLC–Q‐TOF–MS and Network Pharmacology
Source: J Anal Methods Chem. 2026 Feb 11;2026:3075224. doi: 10.1155/jamc/3075224 (PMC12894422; doi:10.1155/jamc/3075224)
Supplement: Supplementary file 1 — Supporting Information Additional supporting information can be found online in the Supporting Information section. [file JAMC-2026-3075224-s001.docx]

**Supplementary Materials to:**

**Characterization and Mechanism Prediction of Active Components in Fuganlin Oral Liquid for Respiratory Tract Infections Using UPLC-Q-TOF-MS and Network Pharmacology**

Mengyue Zhang,^1#^ Feng Han,^2#^ Mingxuan Yang,^1^ Zhishan Ye,^2^ Ying Cui,^1^ Yuefei Wang,^1,3^ Jing Yang,^1*^ Xin Chai,^1,3*^

*^1^ State Key Laboratory of Chinese Medicine Modernization, State Key Laboratory of Component-based Chinese Medicine, Tianjin Key Laboratory of TCM Chemistry and Analysis, Tianjin University of Traditional Chinese Medicine, Tianjin 301617, China*

*^2^ Guangzhou Yipinhong Pharmaceutical Co., Ltd., Guangzhou, Guangdong 510530, China*

*^3^ Haihe Laboratory of Modern Chinese Medicine, Tianjin 301617, China*

*Correspondence: chaix0622@tjutcm.edu.cn (X. Chai)

yangjingoffice@163.com (J. Yang)


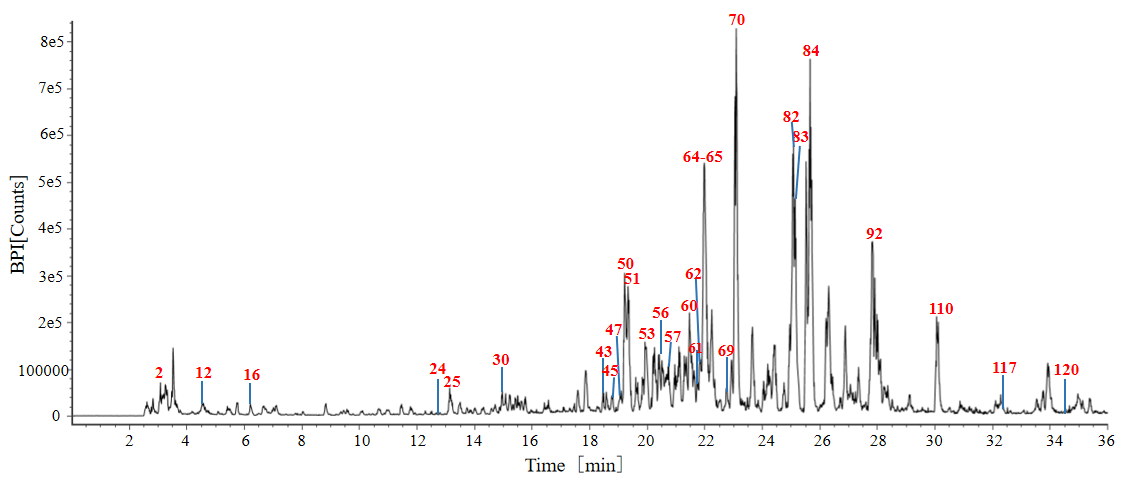

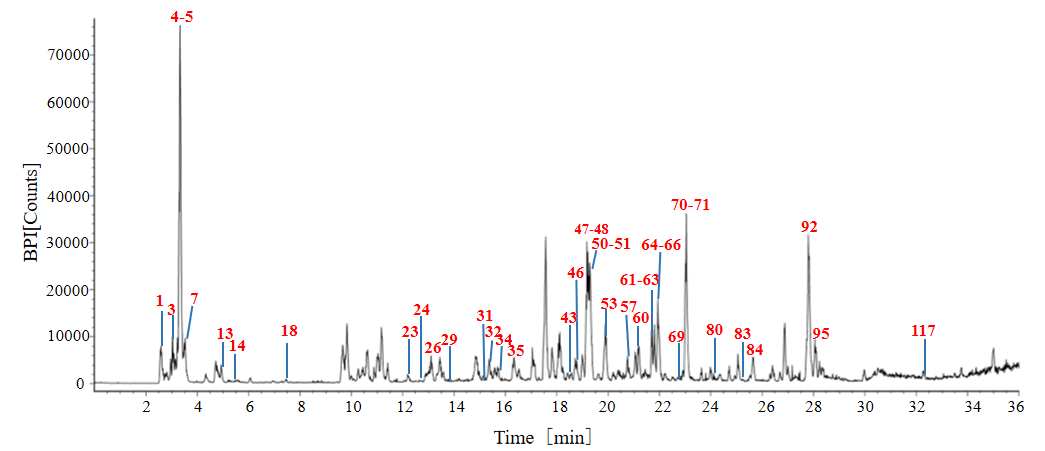


(a1) (a2)


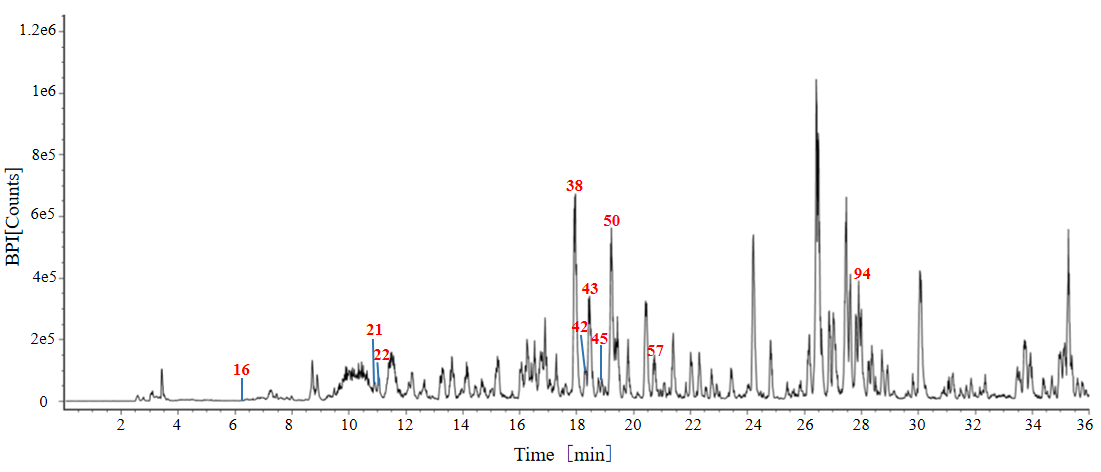

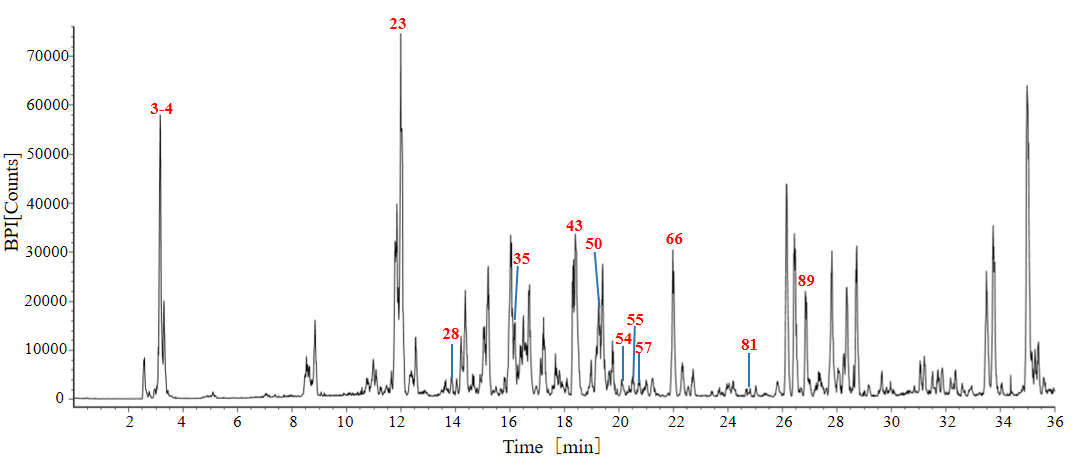


(b1) (b2)


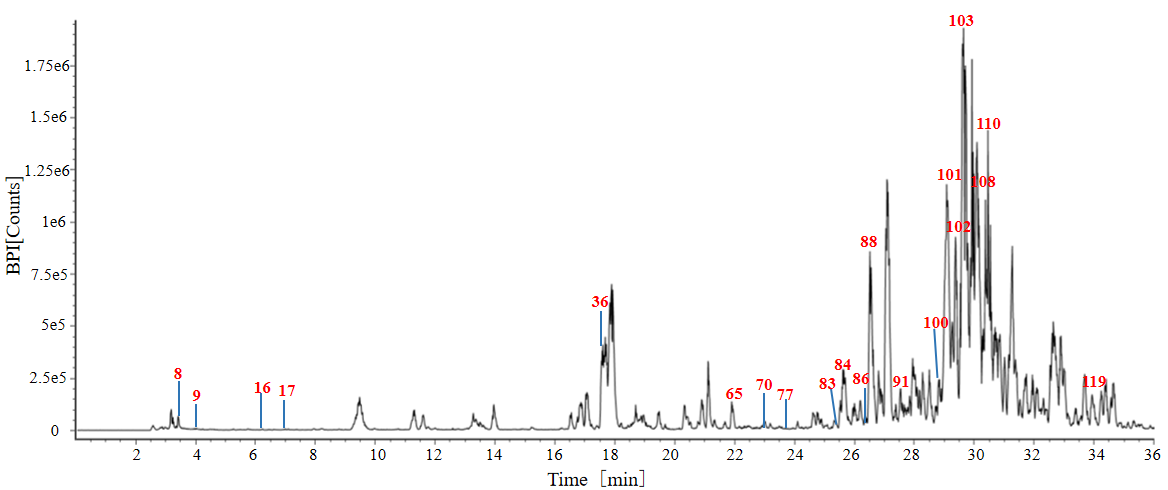

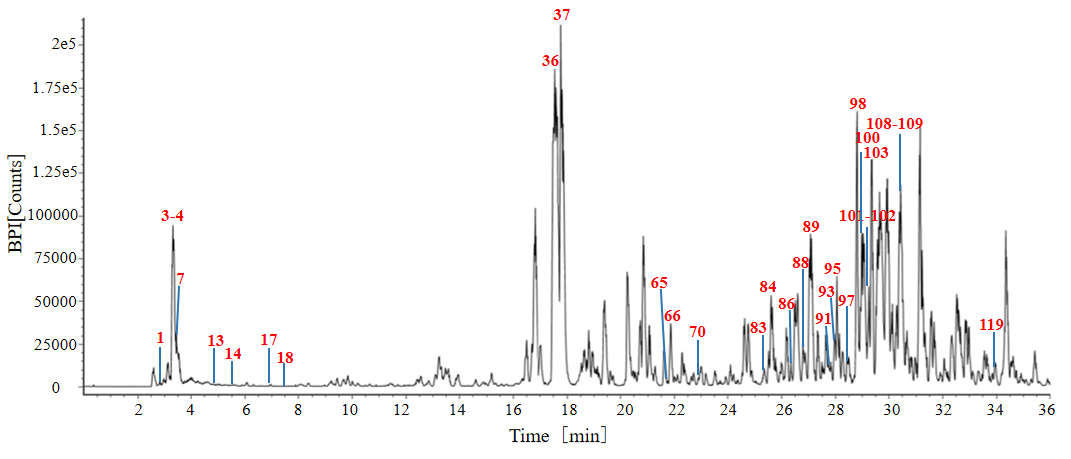


(c1) (c2)


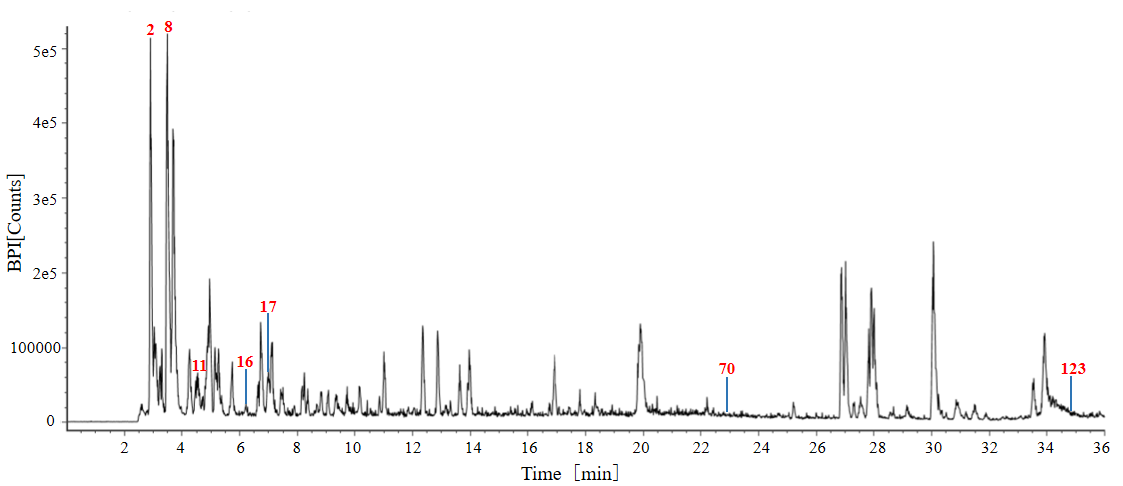

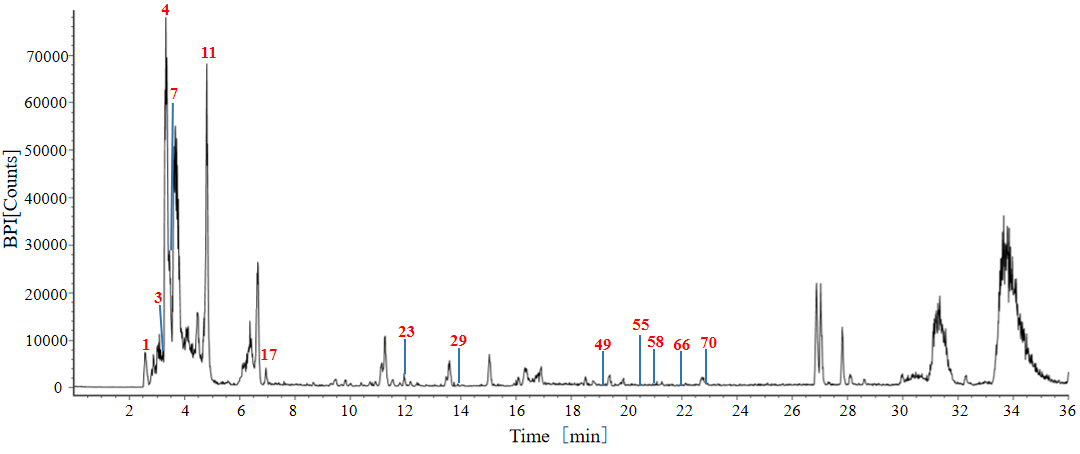


(d1) (d2)


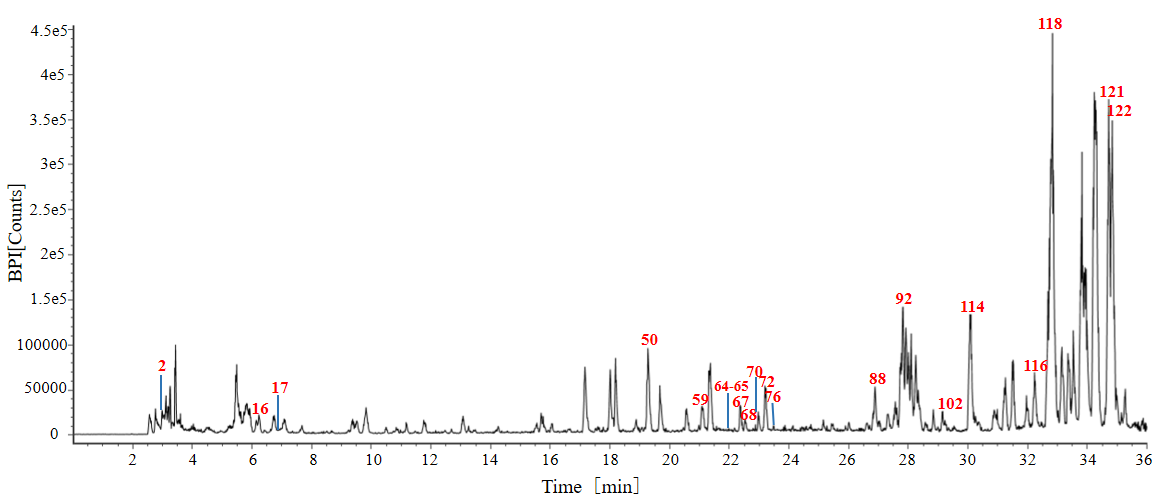

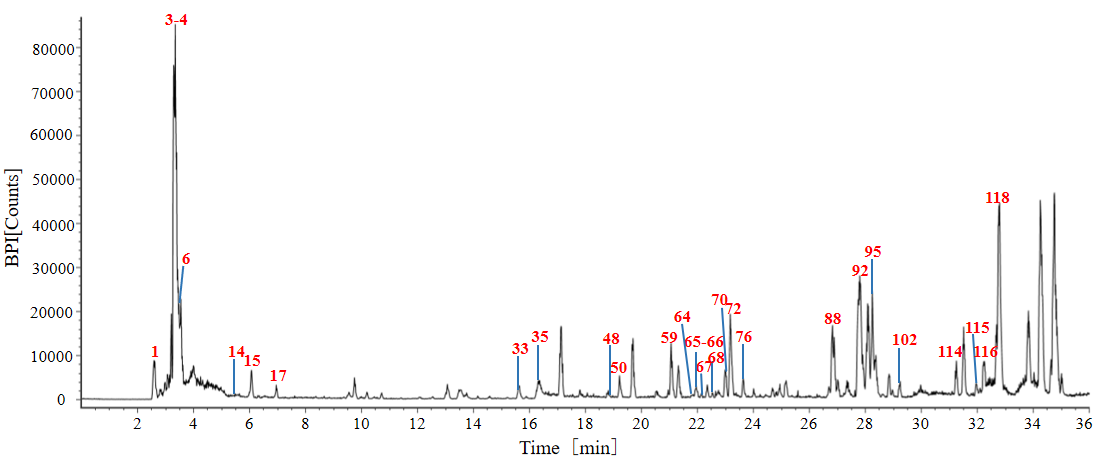


(e1) (e2)


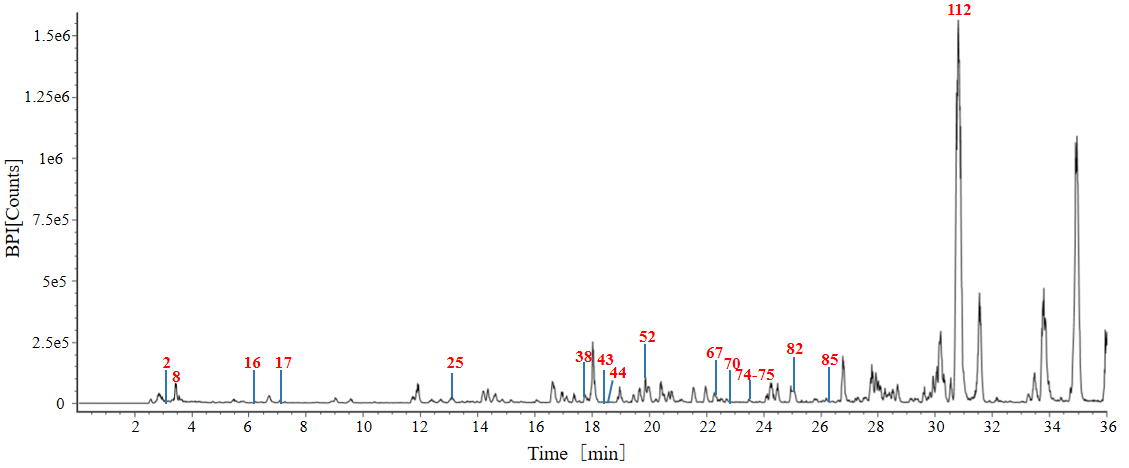

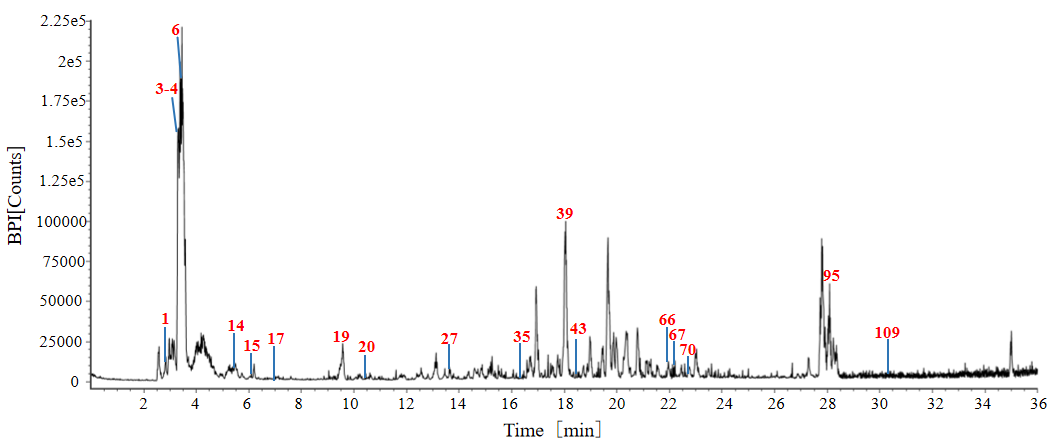


(f1) (f2)


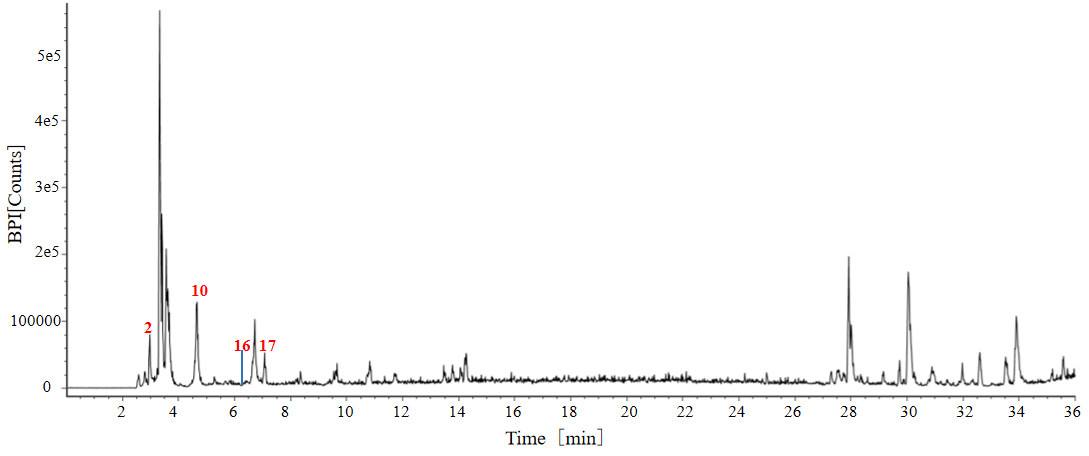

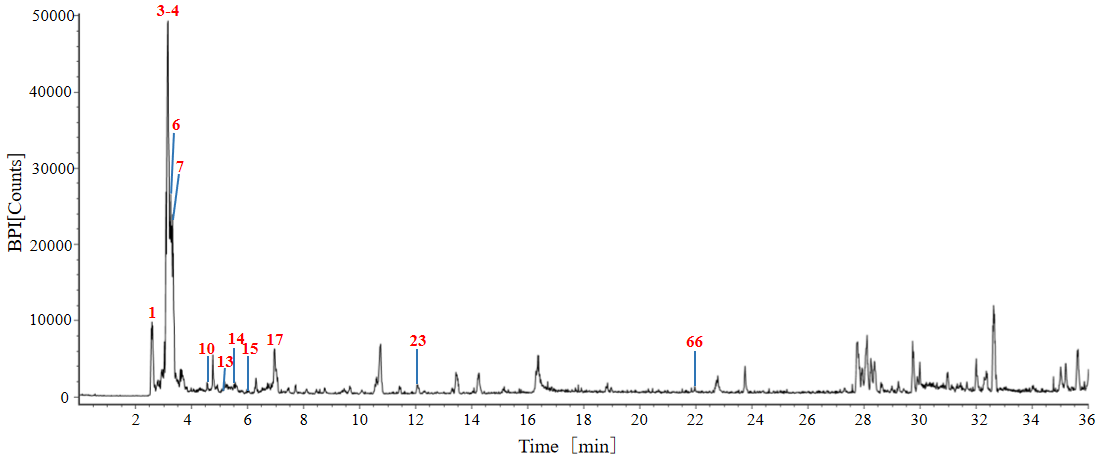


(g1) (g2)


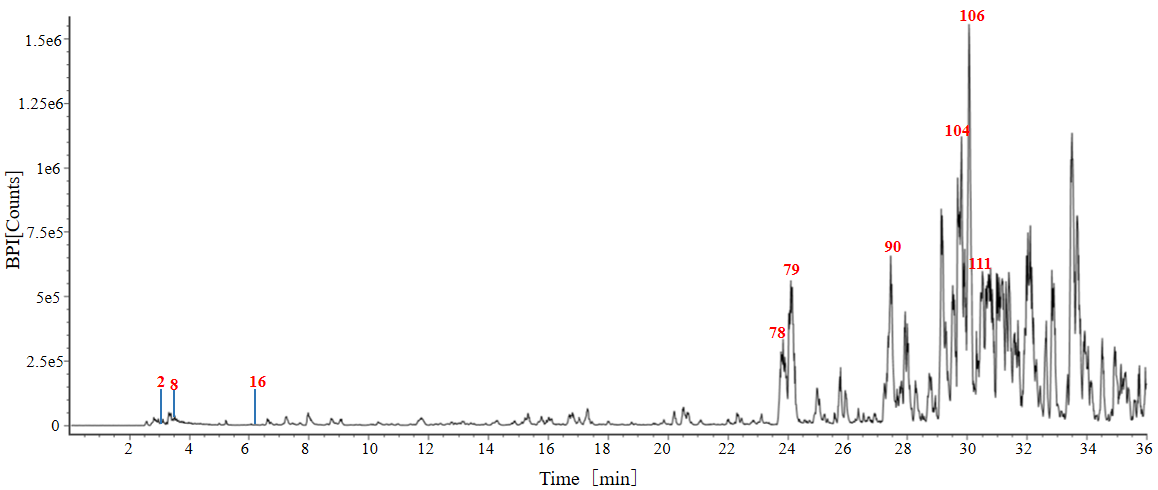

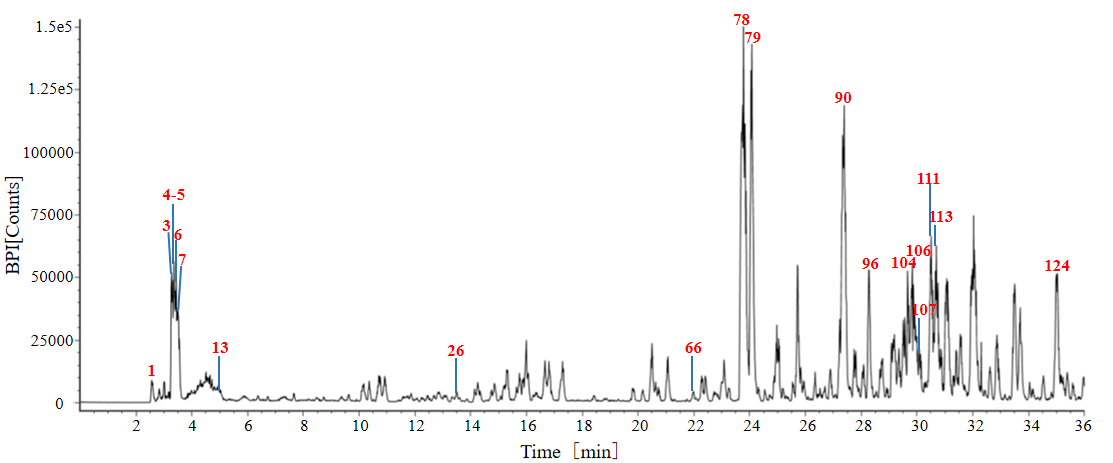


(h1) (h2)


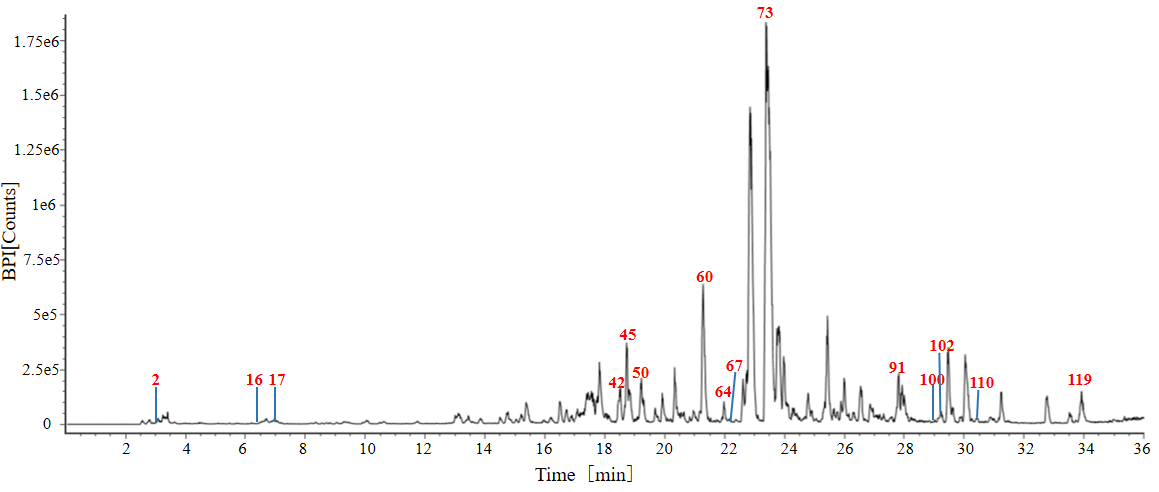

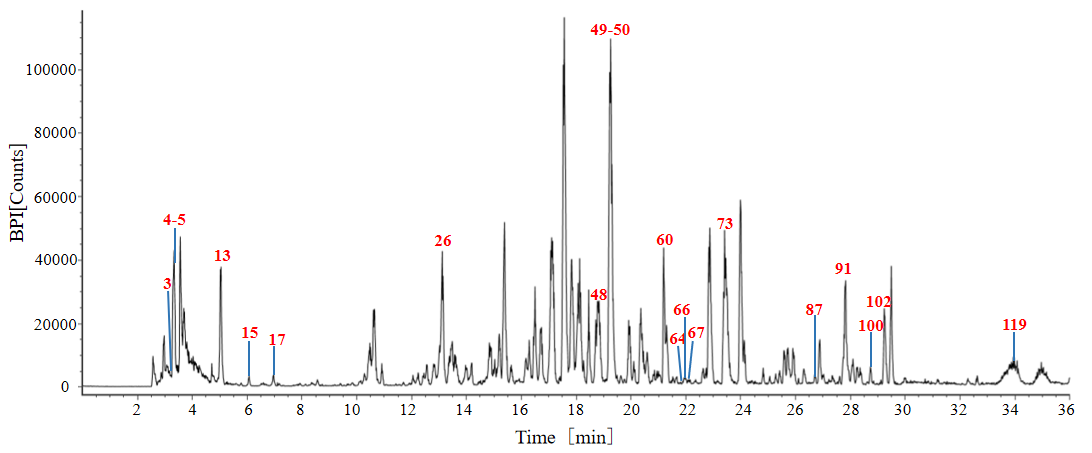


(i1) (i2)


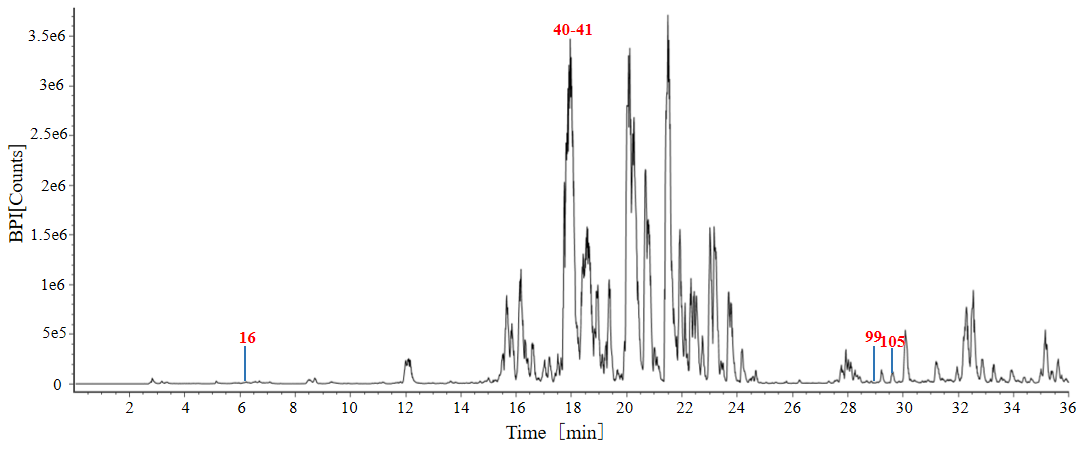

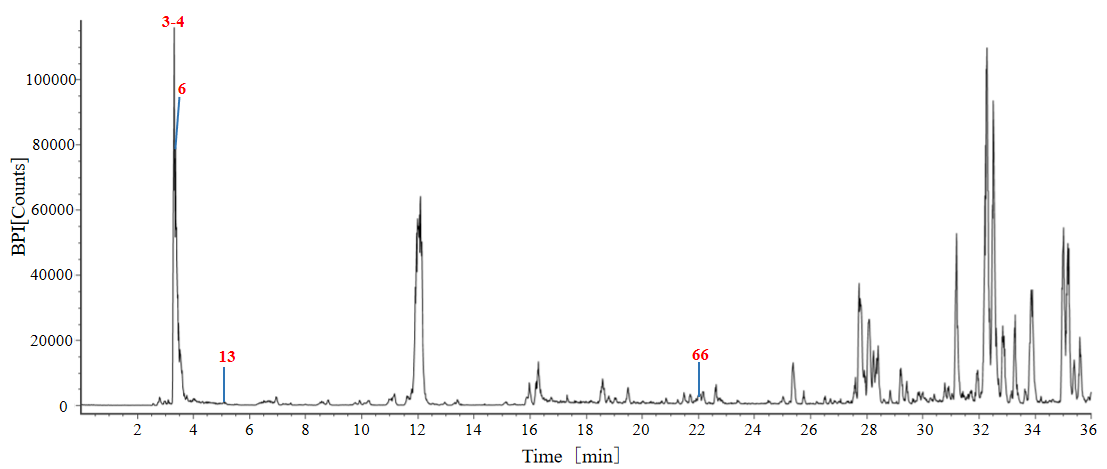


(j1) (j2)

**Figure S1.** The mass spectrum base peak intensity chromatograms of individual herbs from FOL.

Herba Bidentis Pilosae extract in positive (a1) and negative (a2) ion modes, Herba Ephedrae extract in positive (b1) and negative (b2) ion modes, Radix et Rhizoma Glycyrrhizae in positive (c1) and negative (c2) ion modes, Radix Isatidis extract in positive (d1) and negative (d2) ion modes, Radix Astragali extract in positive (e1) and negative (e2) ion modes, Radix Peucedani extract in positive (f1) and negative (f2) ion modes, Edodes Lentinus extract in positive (g1) and negative (g2) ion modes, Radix Panacis Quinquefolii extract in positive (h1) and negative (h2) ion modes, Flos Chrysanthemi Indici extract in positive (i1) and negative (i2) ion modes, Bulbus Fritillariae Thunbergii extract in positive (j1) and negative (j2) ion modes.

**Table S1.** The intersection targets of components in FOL and RTIs.

| CFTR | STAT3 | IL6 | TNF | IL1B | TLR4 | STAT1 |
| --- | --- | --- | --- | --- | --- | --- |
| ALB | EGFR | ELANE | ACE | ACE2 | BRAF | AKT1 |
| IL2 | NFKB1 | PLG | PIK3CA | HMOX1 | MAPK1 | MPO |
| HLA-A | ADA | PIK3CD | PIK3R1 | TERT | MIF | NOS2 |
| ERBB2 | CASP8 | HRAS | IKBKB | CREBBP | PDGFRA | EP300 |
| VEGFA | PTGS2 | NFE2L2 | TYK2 | JAK3 | RET | F2 |
| CCR2 | KIT | FGFR1 | CX3CR1 | GAA | CD81 | PTPRC |
| MT-ND4 | GUSB | PIK3CG | EDNRA | BCL2 | PTPN11 | EIF2AK2 |
| CXCR1 | TLR9 | MMP9 | BMP4 | CXCR2 | RAC1 | VCP |
| JUN | ABCB1 | LCK | SYK | G6PD | MTOR | DPP4 |
| VDR | MET | REN | XIAP | JAK1 | JAK2 | PRF1 |
| CASP3 | CCR3 | PNP | EPHX1 | ADAM17 | PRKCD | F3 |
| CHAT | HIF1A | TTR | CHEK2 | CASP1 | DNMT3B | MMP1 |
| PPARG | MAPK14 | HSP90AA1 | NR3C1 | PRKDC | SLC37A4 | HSPA5 |
| SERPINE1 | CTSL | SRC | GLB1 | CDK4 | CYP3A4 | CDC42 |
| MAPK3 | AR | ALPL | ADRB2 | AGTR1 | MDM2 | TYMS |
| KCNN4 | SELL | MAP2K1 | SETD2 | CCR1 | TK2 | PLEC |
| RAF1 | NOS1 | XDH | SELE | ABL1 | NOS3 | IDO1 |
| GANAB | SLC6A3 | ASAH1 | COMT | NTRK1 | MAPK8 | CYP2C19 |
| ITK | TRPV4 | IGF1R | MAPT | CYP1A1 | SELP | MAN2B1 |
| ALOX5 | FUCA1 | GLUL | ODC1 | FLT4 | PTGS1 | EDNRB |
| BCL2L1 | STAT6 | PON1 | PARP1 | UQCRB | CTSB | ALDH2 |
| FGF2 | MMP2 | HSPA1A | RNASEL | ALK | CFD | PCNA |
| TGFBR1 | LGALS3 | PDGFRB | EPHB4 | BRD4 | MCL1 | VHL |
| AXL | SNCA | FLT1 | ESR1 | CYP2D6 | SIRT1 | RXRA |
| RARA | APP | PLA2G2A | CTSD | MYLK | MB | KDM4C |
| INSR | MANBA | KDR | PPARA | CYP1A2 | HNF4A | GAPDH |
| TYMP | HDAC1 | KMT2A | EZH2 | ANPEP | BCHE | FLT3 |
| EHMT1 | CTSC | RORC | NQO1 | GSK3B | TH | GRB2 |
| FHIT | XPO1 | GLA | MGMT | AKT2 | DNMT1 | GLI1 |
| PDE4A | MMP3 | HPRT1 | PRKCA | APEX1 | HSPA8 | HDAC2 |
| DPP9 | PLA2G4A | KDM5C | PABPC1 | CD38 | RARB | HEXB |
| CDK1 | PLAU | ACHE | ARG1 | NTRK2 | HDAC9 | TEK |
| IGFBP3 | ADCY10 | ABCC1 | PLA2G7 | MAPK10 | TRPV1 | CDC45 |
| SLC5A2 | TACR3 | AKT3 | ENPP1 | LIMK1 | MMP8 | SCN9A |
| PIK3CB | TACR1 | P2RX7 | MMP7 | SIGMAR1 | MAP3K14 | PRKACA |
| MMP14 | CDK2 | TOP2A | NR1H4 | NOX1 | TYR | TP53 |
| SERPINA1 | ATP1A2 | NFKB2 | GSTP1 | LPL | PLAT |  |

**Table S2.** The intersection targets of components-RTIs-anti inflammatory.

| STAT3 | IL6 | TNF | IL1B | TLR4 | ALB | ELANE |
| --- | --- | --- | --- | --- | --- | --- |
| IL2 | NFKB1 | HMOX1 | MAPK1 | MPO | MIF | NOS2 |
| IKBKB | PTGS2 | NFE2L2 | MMP9 | JUN | MMP1 | PPARG |
| MAPK14 | NR3C1 | ALOX5 | PTGS1 | PPARA | PDE4A | MMP3 |

**Table S3.** The intersection targets of components-RTIs-relieving cough and asthma.

| CFTR | STAT3 | IL6 | TNF | IL1B | TLR4 | STAT1 |
| --- | --- | --- | --- | --- | --- | --- |
| ALB | EGFR | ELANE | ACE | ACE2 | AKT1 | IL2 |
| NFKB1 | HMOX1 | MPO | ADA | PIK3CD | TERT | MIF |
| NOS2 | ERBB2 | CASP8 | CREBBP | PDGFRA | VEGFA | PTGS2 |
| NFE2L2 | F2 | CCR2 | CX3CR1 | PIK3CG | EDNRA | BCL2 |
| CXCR1 | TLR9 | MMP9 | CXCR2 | JUN | ABCB1 | SYK |
| DPP4 | JAK1 | CASP3 | CCR3 | EPHX1 | MMP1 | PPARG |
| MAPK14 | HSP90AA1 | NR3C1 | SERPINE1 | SRC | CYP3A4 | MAPK3 |
| AR | ADRB2 | SELL | NOS1 | XDH | SELE | NOS3 |
| IDO1 | ITK | TRPV4 | SELP | ALOX5 | PTGS1 | STAT6 |
| PON1 | PARP1 | ALDH2 | MMP2 | TGFBR1 | FLT1 | PLA2G2A |
| MYLK | CYP1A2 | HDAC1 | NQO1 | PDE4A | MMP3 | PRKCA |
| HDAC2 | PLA2G4A | ARG1 | HDAC9 | PLA2G7 | TRPV1 | MMP8 |
| TACR1 |  |  |  |  |  |  |

**Table S4.** Top 20 key targets and degree of FOL in the treatment of RTIs.

| Target | Degree | Target | Degree |
| --- | --- | --- | --- |
| GAPDH | 194 | TNF | 191 |
| IL6 | 189 | AKT1 | 185 |
| TP53 | 180 | ALB | 174 |
| IL1*β* | 170 | EGFR | 165 |
| STAT3 | 164 | CASP3 | 155 |
| BCL2 | 151 | HIF1A | 150 |
| JUN | 148 | NFKB1 | 145 |
| SRC | 142 | HSP90AA1 | 141 |
| MMP9 | 140 | MAPK3 | 135 |
| ESR1 | 133 | PPARG | 127 |
